# Evolution of the diagnosis of malnutrition in adults: a primer for clinicians

**DOI:** 10.3389/fnut.2024.1169538

**Published:** 2024-02-06

**Authors:** Refaat Hegazi, Anthony Miller, Abby Sauer

**Affiliations:** ^1^Department of Scientific and Medical Affairs, Abbott Nutrition, Columbus, OH, United States; ^2^Department of Food Science and Human Nutrition, University of Illinois at Urbana-Champaign, Champaign, IL, United States

**Keywords:** Malnutrition, diagnosis, definition, GLIM, muscle

## Abstract

During the last two decades, the definition, diagnosis, and management of malnutrition have significantly evolved. Malnutrition is generally defined as deficiencies, excesses, or imbalances in a person’s intake of energy and/or nutrients. While malnutrition is associated with a significantly increased risk of morbidity, mortality, and healthcare cost, it is often underdiagnosed both in healthcare and community settings. One contributing factor is the lack of a consensus on its definition and appropriate diagnostic indicators. In the current article, we review the evolution of frameworks for the diagnosis of malnutrition. Recently published consensuses by prominent clinical nutrition societies have established a trajectory for the uniform global diagnosis of malnutrition. Limiting the use of body mass index (BMI) as a diagnostic criterion while emphasizing the use of muscle mass enables a more consistent and accurate diagnosis of malnutrition in the clinical setting. Guidance for the unified methodology and terminology for diagnosing malnutrition, such as the one proposed in the current article will enable policy makers to systematically address the two faces of malnutrition, starvation- and disease-related malnutrition applicable to both pediatric and adult populations. Policies and programs that could address issues of food insecurity and scarcity as well as early diagnosis and management of disease-related malnutrition will empower better care of community nutrition.

## 1 Introduction

Malnutrition due to illness, poverty, famine, conflict, or natural disasters affects nearly two billion people worldwide (1, 2). Throughout history, hunger and famine have been the most prevalent causes of malnutrition. However, as public health services, food production, and living standards have improved, the definition of malnutrition has become less clear as conditions such as obesity, cachexia, sarcopenia, and micronutrient imbalances have become more prevalent (3–5).

Broadly defined, malnutrition refers to deficiencies, excesses, or imbalances in a person’s intake of energy and/or nutrients in relation to their dietary requirements. Undernutrition, the most classical form of malnutrition, denotes insufficient intake of energy and nutrients to meet an individual’s needs to maintain good health (6). However, undernutrition among adults can develop due to either starvation and/or disease-related inflammation, often due to acute injury or chronic disease (7, 8). Obesity and micronutrient imbalances are also recognized by World Health Organization (WHO) as subsets of malnutrition. Obesity is a paradoxical condition of malnutrition often associated with pathological fat deposition, low muscle mass and a lack of micronutrients, such as copper, zinc, and iodine, despite increased energy intake (9, 10). Deficiency or lack of homeostasis of important micronutrients is another form of malnutrition that has major impacts on everyday performance, intellectual and emotional condition, as well as physical health (11, 12).

The lack of widely accepted global diagnostic criteria to detect patients at nutritional risk who might benefit from nutritional support has been a major concern. The varying terminology and criteria used to define malnutrition make interpreting and comparing prevalence rates and study results difficult. Despite global attempts to define malnutrition, such as consensus statements issued by the American Society for Parenteral and Enteral Nutrition (ASPEN)/The Academy of Nutrition and Dietetics (AND) (13), the European Society of Clinical Nutrition and Metabolism (ESPEN) (14), we still lack a single unambiguous, objective, universally acknowledged consensus definition. However, recently, the Global Leadership Initiative on Malnutrition (GLIM) has provided the basis for a set of globally applicable criteria to diagnose adult undernutrition (15).

The current article details how malnutrition diagnosis has evolved from energy and protein energy malnutrition to etiology-based (starvation-and disease-related malnutrition), over time. The main aims are to provide clarity to both clinicians and community nutrition leaders to combine efforts and enable improved evaluation and monitoring of nutritional status to achieve optimal outcomes.

To guide this review, a virtual meeting of coauthors was held in May 2022 to establish the scope and intent of the publication. The population of interest was established to be adult patients with or at risk of malnutrition in various healthcare settings. Global literature was searched for relevant articles involving the stated population using several tradition engines (PubMed, Google, Cochrane, Embase, and Science Direct) without language or geographic restrictions. The following terms, alone and in combinations, directed the searches: malnutrition, malnutrition risk, diagnosis, adult, hospital, community, guidelines, consensus, outcomes. Applicable publications (61 references) among the hundreds that were identified in multiple literature searches report data regarding the change in malnutrition diagnosis in adult patients over time. Information was assessed to best summarize the evolution of malnutrition diagnosis in the adult population over the past decades, which was the aim of this review.

## 2 Evolution of diagnostic frameworks for diagnosing malnutrition

### 2.1 Marasmus, kwashiorkor, and unspecified protein-calorie malnutrition

For millennia, people have recognized the link between diet and health. In 200 B.C., Hippocrates established the role of nutrition in health when he observed that “the same diet does not suit men in sickness as in health” (16). This simple observation, by today’s standards, was profound in an age when food consumption was thought to provide only a single nutrient to replenish the “innate heat” within each person. Galen, a prominent Greek physician in the post-Hippocrates period, produced the first fundamental work on malnutrition (16). In his book De Marasmo written around 176 A.D., he coined the term “marasmus,” which meant to whither, dry up, waste, or decay (17). Previously thought of as “aging resulting from sickness,” Galen was the first to describe malnutrition, which he split into three types: “due to starvation,” “associated with cold specific to aging,” or “associated with heat specific to fevers” (17). Galen’s seminal work also detailed several physical symptoms associated with malnutrition, which are still diagnostically used today. For example, physical withering is still a key component of several criteria to diagnose malnutrition (13), and the loss of both muscle and adipose tissue mass has recently been identified in diagnostic criteria (18). Importantly, Galen saw that physicians who diagnosed marasmus of old age were able to cure thinness but not wasting with nutritional intervention, reflecting his rudimentary understanding that body fatness is not completely reflective of nutritional status.

Although nutrition played a prominent role in the ancient understanding of health and disease, a comprehensive work on malnutrition would not be completed until the French physician Bernard of Gordon published De Marasmode Secundum Sententiam Galieni in the early 14th century. Gordon devoted much of his work to interpreting the work of Galen from various translations, with an emphasis on defining “**marasmus**,” for which he preferred the definition that implied “drying out” (19). As was common during that time, Gordon relied heavily on analogies to describe the phenomenon he observed. Gordon put forth that marasmus associated with fevers could be compared to an oil lamp and its wick, whereas incineration of the wick reflected the wasting and loss of body mass (19).

Though scientific progress during the next several centuries led to a greater understanding of the chemical basis of nutrition and metabolism, little was written about malnutrition until the late 19th and early 20th centuries, when scientists began to view it as a potentially preventable disease. During this time, meaningful connections were made between nutritional status and disease. For example, it was discovered that an inadequate food supply played a significant role in diphtheria outbreaks (20). The discovery of the link between stored chemical energy and the maintenance of cellular function and structure during the time gave rise to the first diagnoses of malnutrition, which occurred in Europe as early as 1905 (21). As malnutrition became a public health crisis in the United States during the early 19th century, physicians struggled to derive objective measures for its diagnosis. Measurements of height and weight gave way to standardized measurement techniques and criteria meant to make malnutrition diagnosis more consistent (22, 23). However, little progress was made in establishing criteria for diagnosing malnutrition throughout the mid-20th century, although several important studies on the physiology of underlying malnutrition were published (24, 25).

**Figure 1:**
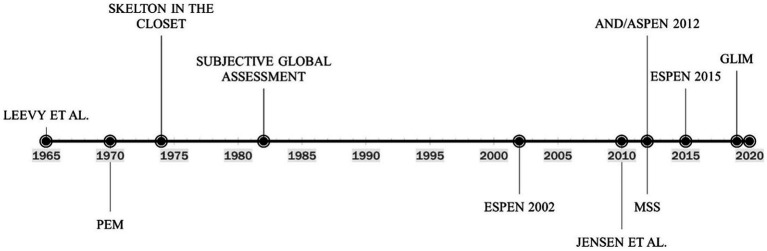
Timeline of the evolution of malnutrition definition and diagnosis. PEM, Protein-energy malnutrition; ESPEN, European society for clinical nutrition and metabolism; AND/ASPEN, the academy of nutrition and dietetics/american society for parenteral and enteral nutrition; MSS, malnutrition-sarcopenia syndrome; GLIM, global leadership initiative on malnutrition.

During the 1960s famine crises in Africa, the WHO brought attention to the medical consequences of starvation (26). They characterized a protein-deficient condition characterized by hypo-albuminemic peripheral edema and ascites, which they termed **kwashiorkor**, and an energy-deficient state characterized by severe weight loss due to fat store depletion, termed marasmus. However, this classification did not turn out to be relevant for recognizing and diagnosing malnutrition in hospitals in Western countries in the late 20th century. The concept of clinical malnutrition, according to our current understanding, was first meaningfully introduced in the 1960s (Figure 1). Malnutrition work during this time often referred to a landmark study by Leevy et al. (27), which found that a substantial percentage of hospitalized patients were micronutrient deficient. Importantly, Leevy’s work foreshadowed increased interest in the clinical diagnosis of malnutrition.

Considering depletion is frequently a combination of protein and energy shortage, the term protein-energy malnutrition (PEM) became widely recognized (28). The clinical parameters used to characterize PEM have evolved, but diagnostic criteria have never been standardized. A variety of biochemical, anthropometric, immunological, and clinical measurements were employed (29). In 1974 (Figure 1), the well-known paper “The Skeleton in the Hospital Closet” by Butterworth (30) along with several other works demonstrated a high prevalence of malnutrition in hospitalized patients (31, 32). The diagnosis of malnutrition began to shift towards the measurement of anthropometric and biochemical markers. Proposed anthropometric markers included body weight (static and change over time), mid-arm muscle circumference (MAMC), and triceps skinfold thickness (TSF) measured in the context of reference standards (33). The use of biochemical markers to diagnose malnutrition was also first described in the 1970s. In 1977, Blackburn et al. (34) proposed the use of serum albumin, transferrin, or total-iron binding capacity as components of nutritional assessment. The concept was reinforced in 1979 when Seltzer et al. (35) recommended that serum albumin be 1 of 2, the other being total lymphocyte count, biochemical parameters for an “instant nutrition assessment”. Subsequently, serum albumin and transferrin became highly utilized markers of malnutrition for hospitalized patients (36, 37). However, clinicians at this time simply considered inflammation to be an identifying symptom of malnutrition and not a causative agent, as would be discovered later.

Although the utilization of biochemical markers of nutrition status continued for decades, their use was challenged as early as 1982, and several authors advocated for focus to shift to the use of patient history and physical examination for malnutrition diagnosis. The Subjective Global Assessment (SGA) was developed in 1982 (Figure 1) to assess patients for malnutrition in the clinical setting. The assessment included a physical exam to identify loss of lean mass and adipose tissue, reflecting an increased appreciation for body composition as it relates to nutritional status. Several clinically-available technologies for measuring body composition also began to emerge and were proposed as alternatives to anthropometric measures (38). The primary body compartment of interest was skeletal muscle (39–41). The term “sarcopenia,” used to describe the age-related loss of muscle, was coined in 1989 (42). In addition to volume changes in skeletal muscle, investigators also became interested in the association between reduced muscle function and nutritional status, with some suggesting that changes in the functional capability of muscle was the most sensitive indicator of malnutrition (43, 44).

In the 1990s, conversation started to shift towards identifying, documenting, and treating malnutrition using a multidisciplinary approach, which spurred the creation of a wide variety of nutrition screening and assessment tools (45) and diagnostic criteria. Renewed focus on the use of suitable biochemical markers of malnutrition resulted in the recommendation of serum transthyretin, otherwise known as prealbumin, as a more sensitive nutrition marker than albumin and transferrin due to its shorter half-life (46).

In 2002 (Figure 1), the European Society for Clinical Nutrition and Metabolism (ESPEN) developed a set of guidelines for nutrition risk screening applicable to a wide range of settings, including community, clinical, and elderly (47). ESPEN recommended the use of the Malnutrition Universal Screening Tool (MUST) in community settings (48), the Nutritional Risk Screening tool (NRS-2002) in clinical settings (49), and the Mini Nutritional Assessment (MNA) for the elderly (50). While these tools shared several diagnostic criteria for malnutrition including body mass index (BMI), weight loss, and disease severity, none of them (screening not diagnostic tools) appreciate criteria such as muscle mass, muscle function, and the presence of inflammation. Furthermore, clinicians relying on the International Classification of Diseases Volume 9 (ICD-9), which was the standard medical coding manual before 2009, were slow to adopt the new screening tools and most often diagnosed malnutrition with code 262 – Other Severe Protein-Calorie Malnutrition, or code 260 – Kwashiorkor (Table 1) utilizing low serum albumin as a primary diagnostic criterion. This oversimplification of malnutrition diagnosis has led to generalized confusion among clinicians, and often misdiagnosis in the countries where ICD codes are heavily relied upon, such as Australia, Canada, China, Germany, South Africa, the United Kingdom, and the United States, among others.

**Table 1:** ICD adult malnutrition codes.

ICD-9	ICD-10
• 260: kwashiorkor • 261: nutritional marasmus • 262: Other severe protein-calorie malnutrition • 263.0: malnutrition of moderate degree • 263.1: malnutrition of mild degree • 263.2: arrested development following protein-calorie malnutrition • 263.8: other protein-calorie malnutrition • 263.9: unspecified protein-calorie malnutrition	• E40: kwashiorkor • E41: nutritional marasmus • E42: Marasmic kwashiorkor • E43: unspecified severe protein-calorie malnutrition • E44.0: moderate protein-energy malnutrition • E44.1: mild protein-energy malnutrition • E45: retarded development following protein-calorie malnutrition • E46: unspecified protein-calorie malnutrition

### 2.2 Etiology-based diagnosis 2010

As methods for clinical analysis of inflammation advanced at the turn of the 21st century, so did the understanding of its role in malnutrition, as both a symptom and causative factor. Clinicians moved away from the use of the combination of kwashiorkor or marasmus and depleted serum albumin to etiology-based diagnosis. In 2010 (Figure 1), Jensen et al. (7) suggested that inflammation-associated catabolism of skeletal muscle is a differentiating factor in the diagnosis of malnutrition and proposed an approach based upon etiology that incorporated the impact of the inflammatory response. Patients with malnutrition and without inflammation could be classified as having “Starvation-Related Malnutrition.” If inflammation is present at a mild to a moderate degree, the patient could be classified as having “Chronic Disease-Related Malnutrition.” If inflammation is present to a severe degree, the patient could be categorized as having “Acute Disease or Injury-Related Malnutrition.”

### 2.3 Malnutrition sarcopenia syndrome 2012

Sarcopenia, defined as loss of muscle mass, strength, and function, can be either primary (age-associated) or secondary (disease, disuse, or undernutrition). Akin to malnutrition, the clinical impact of sarcopenia includes prolonged hospital stay, increased risk of infectious complications, poor wound healing, and mortality. In 2012 (Figure 1), we coined the concept of Malnutrition-Sarcopenia Syndrome (MSS) to highlight the clinical presentation of malnutrition and sarcopenia together in older adults and advocate for the screening, assessment, and treatment of the two conditions concurrently (51). The MSS framework proposes the use of a validated nutrition screening tool, such as MUST, MNA, or NRS-2002 together with the sarcopenia screening tool developed by the European Geriatric Medical Society (EUGMS) Consensus Committee on defining sarcopenia, which employs both gait speed and handgrip strength measurements, although other validated methods of assessing sarcopenic status can be used (51). By assessing patients for both malnutrition and sarcopenia, healthcare practitioners can administer treatments for both conditions, which requires a combination of dietary interventions and muscle strengthening exercises. Additionally, MSS can be diagnosed in both underweight and overweight or obese patients. Furthermore, integrating muscle loss as a diagnostic marker of malnutrition is an important step forward as it highlights the vital role of muscle not only as a structural organ but also for its endocrine, metabolic and immunological functions, and that muscle loss can occur independent of overall body weight (e.g., sarcopenic obesity) making body mass index (BMI) alone as inaccurate marker of overall nutrition health. The updated definition of sarcopenia elevated low muscle strength to the forefront as a primary indicator of sarcopenia and identified poor physical performance as indicative of severe sarcopenia (52). Integrating measures and function in the definition of sarcopenia addresses the technical challenges of availability of muscle mass measurements (e.g., DEXA, MRI, CT) in the hospital setting and permits easier to implement measures of muscle strength and function (e.g., hand-grip strength, chair stand test).

Although MSS has not yet been widely evaluated in health settings, its potential value for predicting poor outcomes in clinical practices has been illustrated in several studies (53, 54). Consistently, other frameworks such as AND/ASPEN have incorporated the measurement of muscle mass as a key diagnostic criterion for malnutrition diagnosis. Similarly, two nutritional screening tools for both conditions have been recently published, Remote—Malnutrition APP (R-MAPP) and PROtocol for NuTritional risk in Oncology (PRONTO) (55, 56).

### 2.4 AND/ASPEN diagnostic criteria 2012

In 2012 (Figure 1), the Academy of Nutrition and Dietetics collaborated with ASPEN to recommend a standardized set of diagnostic characteristics to be used to identify and document adult malnutrition in the clinical setting (13). The consensus statement adopts the approach of Jensen et al., by recommending that patients should first be categorized based on their inflammation status, which can be assessed by a combination of biochemical markers such as serum levels of albumin, prealbumin, C-reactive protein (CRP), or white blood cell count, and clinical signs of inflammation such as fever, hypothermia, or systemic inflammatory responses (e.g., tachycardia, hyperglycemia). Once inflammation status is determined, the clinician can diagnose malnutrition if two or more out of six total characteristics are present. The six characteristics put forward include insufficient energy intake, weight loss, loss of subcutaneous fat, localized or generalized fluid accumulation that may mask weight loss, loss of muscle mass, and diminished functional status as measured by hand grip strength (Table 2). However, measurable numerical guidelines are only put forth for energy intake and weight loss, leaving loss of body fat or muscle mass, fluid accumulation, and reduced grip strength up to the clinician’s interpretation of whether to rate as “mild,” or “moderate to severe.” By incorporating inflammatory status and measures of muscle mass and function, the AND/ASPEN consensus statement represents a significant step forward in the development of a universal framework for the diagnosis of malnutrition.

**Table 2:** Criterion used by recent malnutrition guidelines.

	AND/ASPEN 2012		ESPEN 2015	GLIM 2018	
Severity	Non-severe (moderate) malnutrition	Severe malnutrition	Not included	Moderate malnutrition^c^	Severe malnutrition^c^
Decreased BMI (kg/m^2^)	Not included		Alternative 1^b^ < 18.5 kg/m^2^ Alternative 2^b^ < 20 if <70 yr, or < 22 if ≥70 yr	< 20 if <70 yr, or < 22 if ≥70 yr	<18.5 if <70 yr, or < 20 if ≥70 yr
Decreased energy intake	In the context of acute injury or illness^a^		Not included	≤ 50% of EER for >1 week, or	
	< 75% EER for >7d	≤ 50% EER for ≥5d			
	In the context of chronic illness^a^			any reduction for >2 weeks, or	
	< 75% EER for ≥1 mo	< 75% EER for ≥1 mo			
	In the context of social or environmental circumstances^a^			any chronic GI condition that adversely impacts food assimilation or absorption^d^	
	< 75% EER for ≥3 mo	≤ 50% EER for ≥1 mo			
Weight loss	In the context of acute injury or illness		Alternative 2^b^ 5% over last 3 mo, or 10% indefinite of time	5–10% within the past 6 mo, or 10–20% beyond 6 mo	>10% within the past 6 mo, or > 20% beyond 6 mo
	1–2% in 1 wk	2% in 1 wk			
	5% in 1 mo	5% in 1 mo			
	7.5% in 3 mo	7.5% in 3 mo			
	In the context of chronic illness				
	5% in 1 mo	5% in 1 mo			
	7.5% in 3 mo	7.5% in 3 mo			
	10% in 6 mo	10% in 6 mo			
	20% in 1 y	20% in 1 y			
	In the context of social or environmental circumstances				
	5% in 1 mo	5% in 1 mo			
	7.5% in 3 mo	7.5% in 3 mo			
	10% in 6 mo	10% in 6 mo			
	20% in 1 y	20% in 1 y			
Loss of subcutaneous fat	In the context of acute injury or illness		Not included	Not included	
	Mild	Moderate			
	In the context of chronic illness				
	Mild	Severe			
	In the context of social or environmental circumstances				
	Mild	Severe			
Localized or generalized fluid accumulation	In the context of acute injury or illness		Not included	Not included	
	Mild	Moderate to Severe			
	In the context of chronic illness				
	Mild	Severe			
	In the context of social or environmental circumstances				
	Mild	Severe			
Loss of muscle mass	In the context of acute injury or illness		Alternative 2^b^ FFMI <15 and 17 kg/m2 in women and men, respectively	Mild to moderate deficit^e^	Severe deficit^e^
	Mild	Moderate			
	In the context of chronic illness				
	Mild	Severe			
	In the context of social or environmental circumstances				
	Mild	Severe			
Loss of muscle function	In the context of acute injury or illness		Not included	Not included	
	N/A	Measurably reduced			
	In the context of chronic illness				
	N/A	Measurably reduced			
	In the context of social or environmental circumstances				
	N/A	Measurably reduced			

a Acute or injury or disease-related malnutrition is defined by the presence of a marked inflammatory response. Malnutrition in the context of a chronic illness is defined by inflammation of a mild to moderate degree. Malnutrition in the context of social or environmental circumstances is defined by no inflammatory response.

b ESPEN proposes two alternative ways to diagnose malnutrition; one exclusively based on reduced BMI, and another based on reduced BMI, weight loss, and loss of muscle mass.

c The GLIM guidelines grade severity using three phenotypic criteria: % weight loss, reduced BMI, or reduced muscle mass. Patient must have 1 phenotypic criteria met within the moderate or severe cutoffs.

d Decreased energy intake is not included in GLIM severity grading.

e GLIM provides guidance on the measurement of muscle mass by validated tools such as appendicular lean mass index (ALMI, kg/m^2^) by dual-energy absorptiometry or corresponding standards using other body composition methods like bioelectrical impedance analysis (BIA), CT or MRI, or if not available, anthropometric measurements.

### 2.5 ESPEN diagnostic criteria 2015

In 2015 (Figure 1), ESPEN appointed an international expert group to reach a consensus on a set of generally applicable diagnostic criteria for the diagnosis of malnutrition, independent of etiologic mechanisms and applicable to patients from all clinical settings (14). The guidelines recommend that patients at risk of malnutrition be screened by any validated screening tool. If found to be at risk, the committee recommends the assessment of three variables considered to reflect nutritional status most accurately, namely weight loss, BMI, and free fat mass index (FFMI) (Table 2). The group furthermore provides two alternatives for the diagnosis of malnutrition based on the use of these variables: (1) diagnosis solely based on a BMI, or (2) the combined finding of unintentional weight loss together with either reduced BMI or a low FFMI using sex-specific cut-offs. The inclusion of FFMI reflects the continued appreciation of muscle mass as an important indicator of nutritional status and necessitated the use of modern techniques for body composition analysis. Importantly, the consensus provided numerical cut-offs for each indicator, enabling uniform diagnosis across clinical settings. Regarding inflammation, the consensus group determined that it should be regarded as an etiologic factor rather than a diagnostic feature of malnutrition. This differed from the approach of AND/ASPEN, which considered inflammatory status to be one of the primary differentiators of malnutrition severity and allowed for the use of etiologic variables in the diagnosis of malnutrition.

### 2.6 Global leadership initiative on malnutrition 2018

The discrepancies between malnutrition diagnostic criteria suggested by the two major nutrition societies, ASPEN and ESPEN, called for an opportunity to publish a unified consensus diagnostic criteria set for malnutrition. In 2018 (Figure 1), the Global Leadership Initiative on Malnutrition (GLIM) convened to build a global consensus for diagnostic criteria for malnutrition in adults applicable in diverse global settings (15). Comprised of members from several major global clinical nutrition societies, including ASPEN, ESPEN, the Latin American Federation for Nutritional Therapy (FELANPE), and the Parenteral and Enteral Nutrition Society of Asia (PENSA), the GLIM initiative set its primary aim to “combine clinical accuracy and consistency with a simple implementation that could be applied by nonspecialized healthcare personnel in everyday practice.” As such, the GLIM guidelines represent the most current and comprehensive malnutrition criteria.

Following in the footsteps of the ESPEN 2015 recommendations, the GLIM guidelines recommend an initial screening with a validated screening tool to identify “at risk” status. If a subject is determined to be at risk of malnutrition, it is recommended that clinicians move on to assessment, consisting of malnutrition diagnosis and grading of severity. Malnutrition diagnosis according to GLIM relies on the presence of one etiologic and one phenotypic criterion. Recommended etiologic indicators according to GLIM include reduced food intake (Table 2). Like the ASPEN 2012 guidelines, GLIM also incorporates inflammation as an etiologic criterion, whether related to acute disease/injury or related to chronic disease. The inclusion of inflammation is an important provision that includes new research demonstrating the critical role that systemic inflammation plays in the pathophysiology of malnutrition. To judge whether systemic inflammation is chronic or severe, GLIM recommends the measurement of C-reactive protein (CRP) as a biomarker, although low albumin/prealbumin levels are also included.

Phenotypic criteria include non-volitional weight loss, low BMI, or reduced muscle mass (Table 2). Like ESPEN 2015, GLIM recommends that muscle mass be measured by compositional analysis, such as the analysis of FFMI by dual-energy absorptiometry (DXA). However, recognizing that the availability of analytical equipment varies with geography, physical examination, or standard anthropometric methods such as mid-arm muscle or calf circumference can be used with thresholds adapted to race. Akin to the ASPEN/AND guidelines of 2012, GLIM uses phenotypic criteria to grade the severity of malnutrition as “moderate” or “severe” based on numerical cutoffs of weight loss, BMI, or reduced muscle mass. Even so, the GLIM criteria are less subjective, more clinically intuitive, and include characteristics such as weight loss, muscle mass, and BMI that are more congruent with established ideas of non-severe and severe malnutrition. As malnutrition is not an indexed term in ICD-10, if moderate malnutrition is identified, code E46 (unspecified malnutrition) may be used. If severe malnutrition is documented, code E43 (severe malnutrition) can be employed. It should be noted that, unlike ICD-10, GLIM does not incorporate a mild malnutrition diagnosis (Table 1). Efforts should be made to unify ICD codes with the GLIM guidelines, to ensure that clinicians across the globe diagnose malnutrition by the same criteria.

However, among the criteria included in the GLIM diagnosis, assessment of skeletal muscle mass is least often applied, while BMI continues to be the most applied (57). Recently (Figure 1), the GLIM consortium appointed a working group to provide guidance on the assessment of skeletal muscle mass and the use of muscle function as a diagnostic indicator of malnutrition. The guidance reinforces their original recommendations to utilize a technical approach for measurement of muscle mass [e.g., dual energy x-ray absorptiometry (DXA), bioelectrical impedance analysis (BIA), computerized tomography (CT)] when available, but to use a clinical approach (e.g., anthropometrical measures such as calf circumference or mid-upper arm circumference) if not. Although the working group agreed with the original GLIM guidelines that muscle function cannot serve as a surrogate marker of muscle mass, they leave open the option for physicians to assess for sarcopenia as a phenotypic assessment of malnutrition severity, either as an objective measure of muscle mass or muscle strength.

### 2.7 World Health Organization

According to the WHO, the term malnutrition addresses 3 broad groups of conditions: Undernutrition, which includes wasting (low weight-for-height), stunting (low height-for-age, mainly applicable to the pediatric population), and underweight (low weight-for-age) (58), micronutrient-related malnutrition, which includes micronutrient deficiencies (a lack of sufficient vitamin and mineral intake) or micronutrient excess, and overweight, which includes obesity and diet-related noncommunicable diseases (such as heart disease, stroke, diabetes, and some cancers). While modern frameworks such as GLIM do well to account for malnutrition due to undernutrition and obesity, the WHO framework provides the broadest definition of malnutrition by incorporating malnutrition due to micronutrient abnormalities. While no numerical cutoff values or diagnostic criteria are provided, the WHO framework is an important step for future diagnostic frameworks that account for all the causes of malnutrition.

### 2.8 An integrated malnutrition diagnosis framework

**Figure 2:**
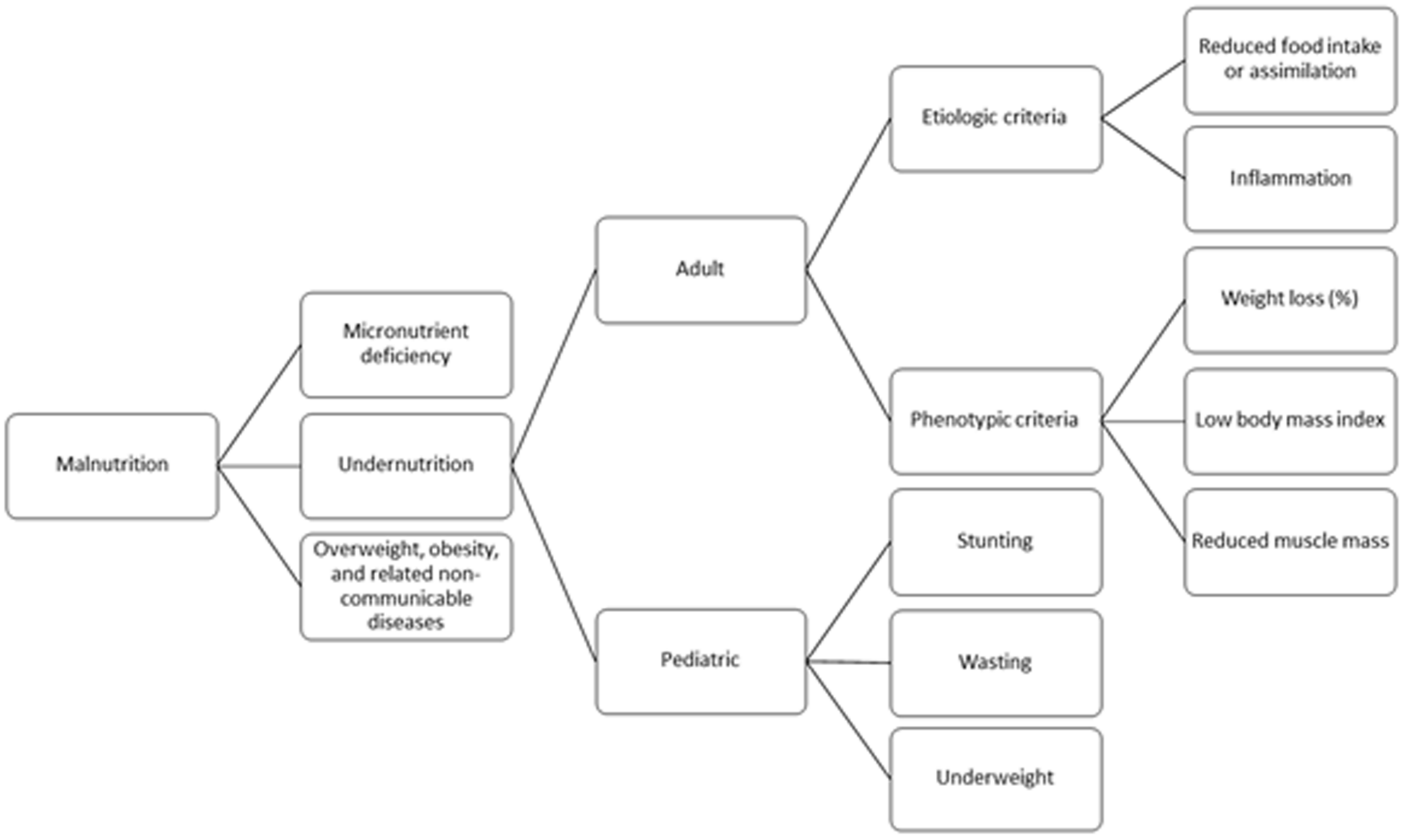
Malnutrition diagnosis algorithm.

Considering the complex history of the definition and diagnosis of malnutrition, we propose that adult malnutrition be defined as a clinical syndrome caused by the imbalance of decreased nutrient intake and increased nutrient demand and characterized by the presence of weight loss, decreased muscle mass and/or function, and/or micronutrient deficiency in the setting of chronic semi-starvation, acute or chronic disease, or obesity. We also propose that the GLIM and WHO frameworks for diagnosing malnutrition be integrated into a single scheme integrating the three forms of malnutrition: undernutrition, obesity and micronutrient deficiency (Figure 2). Integration of loss of muscle (lean) mass (and strength/function) enables clinicians to diagnose malnutrition without solely relying on body weight and BMI (e.g., diagnosis of sarcopenic obesity). According to this proposed and unified framework, symptoms and clinical signs of malnutrition will be assessed as part of clinical examination. Clinicians should routinely assess their patients for history of reduced food intake, weight loss, loss of muscle (lean) mass/ strength/function, micronutrient deficiency, obesity and faltered growth/stunting (in children), Integrating the three forms of malnutrition (triple burden) in clinical care could enable early diagnosis and proper treatment of such a significant disease. Global adoption of an integrated framework with validated methodology and cutoff values for each criterion would enable consistent diagnosis of malnutrition irrespective of geography, improve malnutrition research outcomes and interpretability, and an overall goal of improved patient outcomes.

Although not the main focus of this paper, it is important to note that within the past 5–10 years there have been multiple consensus statements on pediatric malnutrition, and a global integrated framework would be beneficial in this population as well. In 2015, the Academy of Nutrition and Dietetics and American Society for Parenteral and Enteral Nutrition published a recommended standardized set of diagnostic indicators to be used to identify and document pediatric malnutrition (undernutrition) in routine clinical practice (59). The recommended indicators include z scores for weight-for-height/length, body mass index-for-age, or length/height-for-age or mid–upper arm circumference when a single data point is available; and further, when 2 or more data points are available, indicators may also include weight gain velocity (<2 years of age), weight loss (2–20 years of age), deceleration in weight for length/height z score, and inadequate nutrient intake (59). In 2022, the European Society for Paediatric Gastroenterology Hepatology and Nutrition (ESPGHAN) published a position statement on identifying pediatric disease-associated undernutrition (60). This position statement provided an updated descriptive definition of pediatric disease-associated undernutrition as “Undernutrition is a condition resulting from imbalanced nutrition or abnormal utilization of nutrients which causes clinically meaningful adverse effects on tissue function and/or body size/composition with subsequent impact on health outcomes (60).” This position statement recommended that in addition to commonly used criteria for undernutrition such as z score < −2 for weight-for-age, weight-for-length, or body mass index <−2, an unintentional decline of >1in these z scores over time should be considered as an indicator requiring further assessment to establish a diagnosis (60).

## 3 Discussion

Recently, traditional diagnostic indicators of malnutrition such as BMI have faced criticism for their lack of applicability to the population treated in Western hospitals. The use of a low BMI as a phenotypic criterion for malnutrition diagnosis varies significantly by region. Individuals from America are frequently overweight or obese and would need to lose a significant amount of weight before receiving a low BMI designation. However, a low BMI cutoff is still included in the GLIM guidelines because of its ease of measurement and common use in other areas of the globe. On the other hand, the loss of muscle mass is an emerging criterion that is gaining support for inclusion in malnutrition diagnostic guidelines from the clinical nutrition community. Having been included in ASPEN 2012, ESPEN 2015, and GLIM 2018, the methodology for measuring muscle mass and defining what is ‘reduced’ has undergone considerable optimization over the past decade. A primary issue that confronts the use of muscle mass as a global criterion for diagnosing malnutrition is the lack of availability of advanced measurement instruments such as DXA, CT, or BIA. Another limitation is the fact that technology-based methods are often unable to physically accommodate persons with very high body mass. Furthermore, their accuracy is also decreased when used on obese individuals (61). Fortunately, further guidance provided by GLIM points to the anthropometric assessment of muscle mass by calf or mid-arm muscle circumference as being adjustable for persons with high BMI (62). To develop the use of muscle mass as a widely accepted indicator of malnutrition, appropriate cutoff values adjusted for sex and ethnicity must continue to be developed for each methodology, that technology-based methods undergo further refinement and standardization, and, most importantly, that promotion spurs wider clinical awareness of muscle mass as a criterion.

While reduced muscle mass has gained wide acceptance as an indicator of malnutrition, the role of inflammation is less clear. It is commonly accepted that acute or chronic inflammation results in altered body composition and reduced biological function (7, 63), which contributes to malnutrition in several ways, including reduced food intake, increased resting energy expenditure, and muscle catabolism. Based on the work of Jensen et al. (7), modern guidelines for diagnosing malnutrition such as AND/ASPEN 2012 and GLIM 2018 incorporate screening and classification of patients based on their inflammatory status. Although this helps integrate inflammation into the diagnosis of malnutrition, it is not yet clinically well-defined, and biomarkers for detecting the severity of inflammation are not yet agreed upon nor widely utilized as a part of the current clinical practice. In addition, disorders labeled as chronic starvation are not devoid of subtle inflammatory stress.

The present usage of inflammation does not categorize patients with severe illness or acute tissue damage into risk groups to properly decide on the severity of the illness, and therefore the nutritional intervention. For instance, under the current diagnostic framework, all critically ill patients should be classified as having a severe acute injury or disease-related malnutrition. However, pre-admission comorbidities play a major role in determining the clinical outcome of critically ill or acutely injured patients and therefore dictate the appropriate nutritional intervention plan. Two questions need to be further addressed; whether the nutritional requirements of patients with moderate inflammation differ from those of patients with severe inflammation and whether nutrients with anti-inflammatory properties could exert beneficial effects in patients with inflammation-related malnutrition as compared to standard nutrition interventions.

Micronutrient deficiency, frequently overlooked in existing definitions of malnutrition, should be an integral component of malnutrition diagnosis. Patients suffering from both starvation and inflammatory diseases are more likely to be deficient in micronutrients due to inadequate intake and/or increased requirements. Additionally, obese patients have been shown to have deficiencies in almost all micronutrients both before and after bariatric surgery. Clinicians recognize this as the classic combination of macronutrient excess and micronutrient deficiency. Given their essential vital function for normal metabolism, we suggest that micronutrient deficiency become an integral part of a universal malnutrition diagnostic framework. For instance, zinc is a cofactor for the function of several enzymes in glucose, protein, and lipid metabolism, and is crucial for the utilization of glucose by muscle and fat cells. The functions and methods of diagnosis of micronutrient deficiency and treatment have been nicely reviewed by Berger et al. in the 2022 ESPEN micronutrient guideline (64).

An appreciation for the history of the definition and diagnosis of malnutrition, along with continued advancement and unification of the diagnostic frameworks, will provide nutrition experts and clinicians the required foundation to tackle the age-old challenge of properly recognizing and successfully treating malnutrition.

WHO and GLIM diagnostic frameworks are great milestones towards harmonization of the definition and diagnosis of malnutrition. The current proposed framework further builds on these two milestones by integrating the four main forms of malnutrition (adult, pediatric, starvation-related, and disease-related) into one framework. Integrating micronutrient deficiency and obesity-related chronic diseases as integral components of malnutrition diagnosis could further unify efforts to address malnutrition globally. Future research is warranted to validate the cutoffs required for making the diagnosis and classification of malnutrition and coining risk-based therapeutic interventions in different clinical settings. Additionally, the availability of reliable and accessible tools to measure body composition (low muscle/lean mass and high fat to lean mass ratio) and muscle function in clinical settings will enable clinicians to diagnose malnourished patients, tailor treatment plans to their existing phenotype, and monitor effectiveness.

## Author contributions

RH, AM, and AS all contributed to the ideation, creation, and editing of this article. All authors contributed to the article and approved the submitted version.
